# Intensive care unit admission among adults with tuberculosis: a systematic review of clinical characteristics

**DOI:** 10.62675/2965-2774.20260018

**Published:** 2026-09-09

**Authors:** Marjorie Fairuzy Stolarz, Melissa Ferrari Gomes, Gabriel Pavinati, Matheus Cordeiro Marchiotti, Francisco Beraldi de Magalhães, Gabriela Tavares Magnabosco

**Affiliations:** 1 Universidade Estadual de Maringá Undergraduate Program in Nursing Maringá PR Brazil Undergraduate Program in Nursing, Universidade Estadual de Maringá - Maringá (PR), Brazil; 2 Hospital Universitário Regional de Maringá Epidemiological Surveillance Unit Maringá PR Brazil Epidemiological Surveillance Unit, Hospital Universitário Regional de Maringá - Maringá (PR), Brazil.; 3 Faculdades Pequeno Príncipe Curitiba PR Brazil Medical Program, Faculdades Pequeno Príncipe - Curitiba (PR), Brazil.

## INTRODUCTION

Considered a major public health problem, tuberculosis (TB) may progress to severe forms requiring intensive care. Admission to intensive care units (ICUs) reflects complex clinical conditions, frequently associated with respiratory failure, sepsis, and multiple organ dysfunction.^(1)^ In this context, identifying predictors of disease severity is essential to optimize clinical management and to guide more effective therapeutic strategies.

## METHODS

This is a systematic literature review conducted in accordance with the Joanna Briggs Institute guidelines and reported in accordance with the PRISMA checklist. The search was performed in May 2025 across seven databases (PubMed^®^, Cochrane, Scopus, Web of Science, EMBASE, *Biblioteca Virtual em Saúde*, and SciELO), without temporal restriction, including studies published in English, Portuguese, and Spanish, using the following descriptors and Boolean operators: "*Mycobacterium tuberculosis* OR Tuberculosis AND Intensive Care Units OR Critical care AND Adult AND Risk factors OR Associated factors".

The research question was structured using the Population, Concept, and Context (PCC) framework, defined as follows: P - patients with tuberculosis; C - risk factors for hospitalization; and C - intensive care unit. The guiding question was: What are the risk factors for ICU admission among patients with tuberculosis? The review was registered in PROSPERO (CRD42024597749).

## RESULTS

After the initial screening of 1,401 studies, five articles met the eligibility criteria. The studies were conducted in Germany (one), China (one), India (one), and Turkey (two), and were published in English between 2006 and 2021. All adopted a retrospective cohort design, including one multicenter study.

Sample sizes ranged from 35 to 212 individuals with TB admitted to intensive care units (Table 1). Although none of the studies directly addressed the guiding question of this review, the available evidence allowed the analysis to be organized into two main domains: studies describing the reasons for ICU admission among patients with TB, including clinical syndromes, comorbidities, and intensive care support modalities; and studies examining prognostic factors associated with mortality among patients with TB already admitted to intensive care.

**Table 1:** Summary of the characteristics of the studies included in this systematic review

ID	Author	City/ Country	Participants (P) Context (C)	Study design	Main findings
1	Erbes et al.^(2)^	Berlin, Germany	P: 58 individuals C: ICU admission due to active pulmonary tuberculosis	Retrospective single-center cohort	Among the characteristics of patients with TB admitted to the ICU, the following were observed: development of ARDS, decompensation of underlying conditions such as DM, cardiovascular diseases, and COPD, episodes of acute gastrointestinal bleeding, immunosuppression due to HIV infection and the occurrence of opportunistic diseases, electrolyte disturbances, severe anemia, and *delirium* related to alcohol withdrawal and multiple organ dysfunction as one of the main causes of mortality and complications associated with ICU admission
2	Filiz et al.^(3)^	Istanbul, Turkey	P: 35 individuals C: ICU admission due to active tuberculosis and respiratory failure	Retrospective multicenter cohort	Listed as the main causes for ICU admission, individuals requiring intensive care presented with: respiratory failure, sepsis, massive hemoptysis secondary to tuberculosis, extrapulmonary TB involvement (meningeal and pericardial), pre-existing pulmonary diseases (cancer and pneumonia), and alveolar hemorrhage associated with vasculitis. Multiple organ failure is identified as one of the main reasons for requiring intensive care
3	Thomas et al.^(4)^	Vellore, India	P: 212 individuals C: ICU admission due to pulmonary, extrapulmonary, and disseminated tuberculosis	Retrospective single-center cohort	Among the clinical aspects related to ICU admission among individuals with TB, the following were observed: the need for ventilatory support due to pulmonary causes related to TB (ARDS, pneumonia, distributive shock, and pulmonary edema), neurological causes (tuberculous meningitis, seizures, and decreased level of consciousness), and episodes of hemodynamic instability resulting from warm and cold shock states. Secondary factors related to TB included: sepsis, hydrocephalus, bacterial pneumonia, TB-related sequelae, immune reconstitution, pneumothorax/pneumomediastinum, and hyponatremia. Regarding comorbidities associated with the need for intensive care, the following were identified: diabetes mellitus, pre-existing pulmonary diseases (COPD, asthma, and others), autoimmune diseases, cardiovascular diseases, use of immunosuppressive drugs, TB/HIV coinfection, and alcohol use disorder. About factors contributing to mortality among individuals with tuberculosis in the ICU, multiple organ dysfunction played a significant role
4	Tatar et al.^(5)^	Izmir, Turkey	P: 40 individuals C: ICU admission with active pulmonary tuberculosis	Retrospective single-center cohort	Among the reasons for ICU admission among patients with active pulmonary TB, this study identified: the development of respiratory failure requiring ventilatory support, the presence of comorbidities (COPD, diabetes mellitus, and vascular diseases), a history of smoking, and an association with psychiatric disorders. Regarding the main causes of mortality among individuals with tuberculosis in the ICU, the following stand out: multiple organ failure, cardiovascular insufficiency followed by renal failure, and hepatotoxicity-related conditions
5	Wang et al.^(6)^	Jinan, China	P: 82 individuals C: ICU admission due to tuberculosis	Retrospective single-center cohort	Considered key determinants contributing to ICU admission among individuals with tuberculosis, the study identified severe organ failure, development of ARDS, the presence of comorbidities (diabetes mellitus and cardiovascular diseases), and oncologic conditions.

ICU - intensive care unit; TB - tuberculosis; ARDS - acute respiratory distress syndrome; DM - Diabetes mellitus; COPD - chronic obstructive pulmonary disease. Terms reflect the terminology used in the original studies.

Methodological quality was assessed using the Newcastle-Ottawa Scale for cohort studies and the Strengthening the Reporting of Observational Studies in Epidemiology (STROBE) recommendations for observational research. Studies scoring above 70% were considered higher quality, based on evidence from the literature that uses this threshold to classify studies as having good or high methodological quality.^(7)^

## DISCUSSION

Regarding the main reasons for ICU admission among individuals infected with *Mycobacterium tuberculosis*, severe respiratory conditions were consistently reported across studies, including respiratory failure, pneumonia exacerbated by TB, and acute respiratory distress syndrome (ARDS).^(2-6)^ These conditions were frequently associated with the need for invasive mechanical ventilation due to extensive pulmonary damage, often aggravated by pre-existing respiratory diseases such as asthma and bronchiectasis, particularly chronic obstructive pulmonary disease (COPD).^(2,4,5)^ These findings are consistent with current scientific literature, which highlights the severity of respiratory involvement in TB within intensive care settings. Among factors associated with mortality, multiple organ dysfunction^(2-6)^ emerged as a relevant contributor, with reported incidence ranging from 3.4%^(2)^ to 48.6%^(3)^ across the included studies. Although considered relatively uncommon, it represents a severe complication requiring intensive care management^(3)^ and is associated with poor prognosis, particularly in the context of secondary sepsis. The literature indicates that its occurrence is more frequent in cases of disseminated TB and among immunocompromised individuals, especially those with TB/HIV coinfection, which has been identified as another factor increasing mortality risk. This condition may lead to cardiovascular, hepatic, renal, and respiratory failure, often necessitating hemodynamic and renal support, as well as more complex therapeutic approaches.^(8-10)^ Severe immunosuppression further predisposes individuals to disseminated disease, increasing clinical severity, impairing host immune response, and heightening susceptibility to opportunistic infections.^(11)^

Despite these findings, there remains a scarcity of studies specifically addressing predictors of ICU admission among individuals with TB. This gap in the literature limits the development and refinement of public health policies and care models aimed at preventing disease progression to critical stages, thereby reducing the need for intensive care and mitigating the associated psychosocial and economic impacts.

## Data Availability

The contents underlying the research text are included in the manuscript.
